# How We Interpret Thrombosis with Thrombocytopenia Syndrome?

**DOI:** 10.3390/ijms25094956

**Published:** 2024-05-01

**Authors:** Shinya Yamada, Hidesaku Asakura

**Affiliations:** Department of Hematology, Kanazawa University Hospital, Takaramachi 13-1, Kanazawa City 920-8640, Ishikawa, Japan; abacus3shinya@gmail.com

**Keywords:** thrombosis with thrombocytopenia syndrome, heparin-induced thrombocytopenia, vaccine-induced immune thrombotic thrombocytopenia, paroxysmal nocturnal hemoglobinuria, antiphospholipid antibody syndrome, thrombotic microangiopathy, disseminated intravascular coagulation, thrombotic thrombocytopenic purpura, hemolytic uremic syndrome

## Abstract

Platelets play an important role in hemostasis, and a low platelet count usually increases the risk of bleeding. Conditions in which thrombosis occurs despite low platelet counts are referred to as thrombosis with thrombocytopenia syndrome, including heparin-induced thrombocytopenia, vaccine-induced immune thrombotic thrombocytopenia, paroxysmal nocturnal hemoglobinuria, antiphospholipid syndrome, thrombotic microangiopathy (TMA), and disseminated intravascular coagulation. TMA includes thrombotic thrombocytopenic purpura, Shiga toxin-producing *Escherichia coli*-associated hemolytic uremic syndrome (HUS), and atypical HUS. Patients with these pathologies present with thrombosis and consumptive thrombocytopenia associated with the activation of platelets and the coagulation system. Treatment varies from disease to disease, and many diseases have direct impacts on mortality and organ prognosis if therapeutic interventions are not promptly implemented. Underlying diseases and the results of physical examinations and general laboratory tests as part of a thorough workup for patients should promptly lead to therapeutic intervention before definitive diagnosis. For some diseases, the diagnosis and initial treatment must proceed in parallel. Utilization of not only laboratory tests but also various scoring systems is important for validating therapeutic interventions based on clinical information.

## 1. Introduction

Usually, a marked decrease in platelet count is associated with an increased frequency of bleeding. For example, in idiopathic thrombocytopenic purpura or immune thrombocytopenia (ITP), the mortality risk is 4.2 times higher (95% confidence interval, 1.7–10.0) in the group with platelet counts ≤30,000/μL than in the group with platelets >30,000/μL [1], and the risk of fatal hemorrhage has been reported to increase dramatically [1,2]. On the other hand, a marked increase in platelet count increases the frequency of thrombosis. For example, in essential thrombocythemia (ET), an increased risk of thrombosis has been reported for platelet counts exceeding 1,250,000/μL [3].

However, the opposite pathology also exists. That is, bleeding when the platelet count is increased and thrombosis when the platelet count is decreased. In ET, bleeding is reportedly increased when the platelet count exceeds the upper limit of normal compared with patients within normal platelet counts (hazard ratio 3.7; 95% confidence interval, 1.7–8.2, *p* = 0.001) [3]. One reason for this is a decrease in the von Willebrand factor (VWF) due to adsorption by excess platelets [4]. In addition, the proteolytic enzymes a disintegrin and metalloproteinase domain-containing protein (ADAM) 10 [5], and ADAM17 [6] are secreted by excessively increased platelets, resulting in the degradation of high-molecular weight-VWF (HMW-VWF) multimer [7]. Another possible mechanism is the excessive cleavage of high-molecular weight-VWF multimer by a disintegrin-like and metalloproteinase with thrombospondin type 1 motifs 13 (ADAMTS13) [8], which occurs when excess platelets increase intravascular shear stress, causing the HMW-VWF multimer structure to extend and facilitating cleavage by ADAMTS13.

**Figure 1:**
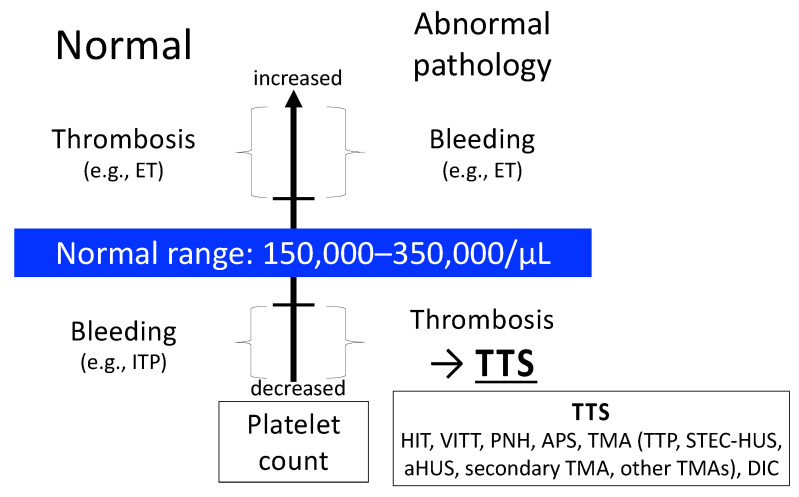
Platelet count abnormalities and clinical features. Normally, an increased platelet count increases the risk of thrombotic events, while a decreased platelet count increases the risk of bleeding. However, ET is a disease that presents with bleeding symptoms despite an increased platelet count. Diseases that present with thrombosis despite low platelet counts are termed TTS, which include HIT, VITT, PNH, APS, TMA, and DIC. TMA is classified as TTP, STEC-HUS, aHUS, secondary TMA, and other TMAs. Abbreviations: ET, essential thrombocythemia; ITP, idiopathic thrombocytopenic purpura/immune thrombocytopenia; TTS, thrombosis with thrombocytopenia syndrome; HIT, heparin-induced thrombocytopenia; VITT, vaccine-induced immune thrombotic thrombocytopenia; PNH, paroxysmal nocturnal hemoglobinuria; APS, antiphospholipid antibody syndrome; TMA, thrombotic microangiopathy; TTP, thrombotic thrombocytopenic purpura; STEC-HUS, Shiga toxin-producing *Escherichia coli*-associated hemolytic uremic syndrome; aHUS, atypical hemolytic uremic syndrome; DIC, disseminated intravascular coagulation.

Various conditions can cause thrombosis despite low platelet counts, including heparin-induced thrombocytopenia (HIT), vaccine-induced immune thrombotic thrombocytopenia (VITT), paroxysmal nocturnal hemoglobinuria (PNH), antiphospholipid antibody syndrome (APS), thrombotic microangiopathy (TMA), and disseminated intravascular coagulation (DIC) [9]. TMA includes thrombotic thrombocytopenic purpura (TTP), Shiga toxin-producing *Escherichia coli*-associated hemolytic uremic syndrome (STEC-HUS), and atypical HUS (aHUS) [10]. Platelet counts and clinical features are summarized in Figure 1.

This review discusses the category of diseases presenting with thrombosis despite a low platelet count with reference to disease etiology, diagnostic methods, laboratory characteristics, and treatment. Approaches to differentiating these diseases in daily clinical practice are examined.

## 2. Characteristics of Thrombosis with Thrombocytopenia Syndrome

Each of the thrombosis with thrombocytopenia syndrome (TTS) mentioned above is reviewed in terms of etiology, diagnostic methods, characteristics of laboratory data, and treatment.

### 2.1. HIT

Antibodies against the platelet factor 4 (PF4)/heparin complex (HIT antibodies) are involved in the development of HIT [11]. The presence of heparin and PF4 at moderate concentrations causes structural changes in PF4, exposing antigenic determinants on the PF4 surface and leading to the production of HIT antibodies by B lymphocytes [12]. The HIT antibody forms an immune complex with the PF4/heparin complex, which activates platelets by binding to platelet membrane FcγRIIA receptors [13] and releasing microparticles leading to coagulation activation [14]. HIT antibodies also bind to vascular endothelial cells and monocytes, increasing expression levels of tissue factor in vascular endothelial cells and monocytes, which in turn promote thrombus formation and cause thromboembolism [15,16,17]. At the same time, excessive activation of platelets causes consumptive thrombocytopenia. There is also a pathological condition called autoimmune HIT or spontaneous HIT, in which antibodies similar to HIT antibodies are produced in response to surgery, trauma, or infection even in the absence of heparin exposure [18].

In clinical practice of HIT, it is important to make a clinical diagnosis and initiate treatment before a definitive diagnosis is performed by testing. The 4Ts score is used to calculate a pretest score for the presence of HIT: thrombocytopenia (platelet count decrease); timing of platelet count decrease; thrombosis; and other causes of platelet count decrease. If the score is high and HIT is strongly suspected clinically, treatment as described below must be started without waiting for a definitive diagnosis [19,20]. The presence of HIT antibodies is then confirmed by latex agglutination or a chemiluminescence immunoassay. Immunological assays offer nearly 99% sensitivity and are highly useful for excluding HIT diagnoses, but specificity is not high, and even with a positive result, HIT is difficult to confirm [21]. For a definitive diagnosis, functional assays are used to assess whether HIT antibodies can activate platelets [22]. However, few facilities are able to implement functional measurement methods and strict accuracy control is required, making such assays difficult to implement in daily clinical practice.

Platelet counts show a consumptive decrease in HIT. The presence of HIT alone does not affect prothrombin time (PT), activated partial thromboplastin time (APTT), or fibrinogen level, but these test values may change due to the use of heparin or as a complication of DIC. As reflections of excessive coagulation activation and thrombus formation, levels of fibrin/fibrinogen degradation products (FDP), D-dimer and the coagulation activation marker thrombin–antithrombin complex (TAT) significantly increase [23]. Diagnosing or excluding HIT based on coagulation and fibrinolysis testing alone is difficult, necessitating an appropriate combination of the 4Ts score with immunoassays and, where possible, functional assays.

Platelet activation inhibition and thrombin activity inhibition are two key points for the treatment of HIT. Treatments include discontinuation of heparin, intravenous immunoglobulin (IVIg), and plasma exchange [24,25,26]. IVIg aims to inhibit the binding of HIT antibodies to platelets by taking up binding sites in advance [27]. Plasma exchange is expected to remove HIT antibodies, but evidence remains insufficient. Argatroban is the treatment of choice for achieving thrombin activity inhibition [28,29], but danaparoid and fondaparinux may also be used [30,31]. Direct oral anticoagulants (DOACs) are recommended as anticoagulants in the chronic phase as these drugs carry a lower risk of bleeding than warfarin, but the risk of new thrombus formation is similar [32]. In about half of HIT patients, HIT antibodies become negative in about 50–85 days [33], so the use of DOACs is often completed in approximately 3 months. Re-administration of heparin may cause the recurrence of HIT [34,35,36,37], but heparin can reportedly be safely administered after negative results are obtained for HIT antibodies [38,39,40]. Re-administration of heparin may thus be permissible in such limited cases.

### 2.2. VITT

By March 2021, shortly after the ChAdOx1 nCoV-19 adenovirus-vectored vaccine against severe acute respiratory syndrome coronavirus 2 (SARS-CoV-2) was administered in Norway, Germany, Austria, and the United Kingdom, serious side effects were reported. After vaccination with ChAdOx1 nCoV-19, thrombosis in unusual sites such as the splanchnic veins, portal vein, thoracic vertebral veins, and basivertebral veins and decreased platelet counts have been reported one after another, with this presentation now called VITT [41,42,43,44] or vaccine-induced prothrombotic immune thrombocytopenia or vaccine-induced TTS [45]. TTS occurring after vaccination with an adenovirus vector-type vaccine (e.g., ChAdOx1 nCoV-19 or Ad26.COV2.S vaccine) against SARS-CoV-2 is called VITT. However, mRNA-type vaccines (mRNA-1273 [46,47,48,49] and BNT 162b2 [50,51]) and even vaccines against human papillomavirus [52] have also been reported to cause VITT. Regardless of the type of vaccine, attention should be paid to signs of VITT that develop after vaccination [52].

VITT caused by adenovirus vector vaccines results from the formation of autoantibodies (PF4/DNA complex antibodies: VITT antibodies) against a complex consisting of free DNA present in the vaccine and platelet factor 4 (PF4). The Fc portion of the VITT antibody is thought to bind to the FcγRIIA receptor on the platelet membrane, causing platelet activation and aggregation [41]. Furthermore, microparticles containing tissue factor are released from activated platelets, promoting thrombus formation and the development of thrombosis [53]. The VITT antibody binds to heparan sulfate and chondroitin sulfate on monocytes and the vascular endothelium and enhances the expression of tissue factor, leading to the overproduction of thrombin and overactivation of coagulation [41].

Five items have been proposed as diagnostic criteria for VITT [54]. Depending on the number of items met, VITT is classified as “Definite VITT”, “Probable VITT”, “Possible VITT”, or “Unlikely VITT”. These five items are:

- Onset approximately 5–30 days after SARS-CoV-2 vaccination;
- Presence of thrombosis;
- Decrease in platelet count (<150,000/μL);
- D-dimer increase to ≥4 μg/mL;
- Anti-PF4 antibody-positive results from enzyme-linked immunosorbent assay (ELISA) assay.

One report summarized the results of coagulation and fibrinolysis tests from 220 cases of VITT [54]. The median platelet count was 47,000/μL (interquartile range (IQR): 28,000–76,000/μL), the median PT was 13 s (IQR: 10–14 s), the median APTT was 29 s (IQR: 22–30 s), the median fibrinogen level was 220 mg/dL (IQR: 120–310 mg/dL), and the median D-dimer level was 12 μg/mL (IQR: 4–18.5 μg/mL). According to that report, lower platelet count, lower fibrinogen level, and higher D-dimer level were all associated with poorer prognosis. Another report also indicated that lower fibrinogen level and lower platelet count represented poor prognostic factors in VITT [55]. The FAPIC score, named after the initials of the items, has been proposed as a scoring system to predict the prognosis of VITT, using five items: fibrinogen <150 mg/dL; age <60 years; platelet count <25,000/μL; intracerebral hemorrhage; and cerebral venous thrombosis [55].

A characteristic feature of coagulation and fibrinolysis tests for VITT is that while PT and APTT are almost normal, fibrinogen levels are low and D-dimer levels are high [56]. One disease showing a similar profile of coagulation and fibrinolysis test findings is enhanced-fibrinolytic-type DIC [57,58,59]. In enhanced-fibrinolytic-type DIC, marked elevations are seen in both plasmin-α_2_-plasmin inhibitor complex (PIC), a fibrinolytic activation marker, and TAT, a coagulation activation marker, and severe bleeding symptoms occur due to excess fibrinolysis of hemostatic thrombi [57,58,59]. VITT complicated by DIC shows a poor prognosis [56], and most deaths in VITT are due to bleeding [55]. At least 45% of VITT cases are complicated by DIC, and elevated PIC is observed in some cases [60]. In other words, enhanced-fibrinolytic-type DIC may also contribute to the bleeding that is thought to be the main cause of death in VITT.

Safe treatment for VITT involves high-dose immunoglobulin therapy to suppress Fc receptor-mediated platelet activation [61,62,63]. PF4/DNA complex antibodies are also thought to bind to PF4/heparin complexes and induce platelet activation, so the use of heparin for anticoagulant therapy is initially avoided [41,42,64,65]. The epitope by which the VITT antibody binds to PF4 was shown to differ from the epitope to which the HIT antibody binds [66]. Heparin administration may actually inhibit the binding between PF4 and VITT antibodies; thus, heparin administration may improve therapeutic outcomes [66]. In fact, the use of heparin has been reported as safe and effective [54,67]. Other anticoagulant therapies considered useful for VITT include argatroban, DOACs, fondaparinux, and danaparoid [68]. Warfarin induces protein C deficiency and may worsen thrombosis. In addition, the use of warfarin for DIC may instead exacerbate DIC and promote bleeding [69,70]. Warfarin should therefore be avoided in VITT complicated by DIC. In addition, the effectiveness of steroids [54], plasma exchange [54,71,72,73], inhibition of complement [44,74], and inhibitors of the bruton kinase pathway [75] remain controversial and require further investigation. Worldwide, argatroban is the anticoagulant therapy most commonly used for VITT. However, there are concerns that some patients with VITT may be using argatroban without being aware of the complication of enhanced-fibrinolytic-type DIC, inducing fatal bleeding. In fact, a clinical trial of argatroban for DIC being conducted in Japan more than 30 years ago was suspended due to major bleeding events. From the current perspective, argatroban in that study seems likely to have been administered to patients with enhanced-fibrinolytic-type DIC. For VITT complicated with enhanced-fibrinolytic-type DIC, one option may be to use argatroban at a reduced dose in combination with nafamostat, which has weak anticoagulant and strong antifibrinolytic effects [56,76].

### 2.3. PNH

PNH results from mutations in the PIG-A gene, which is required for synthesis of the glycosylphosphatidylinositol (GPI) anchor in hematopoietic stem cells. The disease is caused by a deficiency of GPI-anchored complement regulators CD55 and CD59 on the erythrocyte membrane, resulting in enhanced complement activity on erythrocytes and intravascular hemolysis. When complement activation is temporarily increased by infection, hypovolemia due to sleep [77,78], pregnancy, surgery, or excessive vitamin C intake [79], hemolysis is aggravated and results in hemolytic attacks with gross hemoglobinuria. This hemolysis also results in the release of large amounts of free hemoglobin, which lower nitric oxide in the blood, causing smooth muscle spasm, dysphagia, abdominal pain, erectile dysfunction [80], and acute and chronic renal failure due to hemosiderin deposition [80,81,82]. In addition, decreased nitric oxide [80] and complement activation leads to platelet aggregation and activation, resulting in thrombus formation and a consumptive reduction in platelet count. Complement activation may result in the increased release of microparticles from abnormal red blood cells and platelets [83] and decreased nitric oxide due to trapping of free hemoglobin [80], leading to endothelial damage and thrombus formation. Around 17–40% of PNH patients have clinical arteriovenous thrombosis with symptoms [84,85], and this thrombosis accounts for 40–67% of PNH-related deaths [85,86,87].

No reports appear to have discussed the characteristics of coagulation test results in PNH. Early diagnosis and therapeutic intervention are desirable because initial thrombotic events increase the risk of death 5- to 10-fold [85], thrombotic events occur a median of 2.3 years after diagnosis [88], and the risk of thrombosis increases with increasing size of the PNH clone [89]. Haptoglobin should also be administered during hemolytic attacks to prevent thrombotic complications [90]. In addition, data from before the advent of complement inhibitors suggest that warfarin was more effective than other anticoagulants in preventing thrombosis [91], but since the advent of the complement inhibitor eculizumab, complement inhibition itself has significantly reduced thrombotic events more than warfarin [92]. Anticoagulation therapy is usually initiated for patients who develop thrombosis, but no evidence exists regarding which anticoagulant is better.

### 2.4. APS

Antiphospholipid antibodies (aPLs) are autoantibodies that recognize phospholipids and phospholipid/phospholipid binding protein complexes. In vitro, aPL binds to phospholipids, which are reagent components of the method for determining APTT, inhibiting the coagulation reaction and thus prolonging APTT [93]. On the other hand, the presence of aPLs in vivo promotes coagulation and causes thrombosis. As a mechanism for causing thrombosis, antiphospholipid antibodies inhibit the activation of protein C, inhibit β2-glycoprotein-I (β2-GPI) that suppresses phospholipid-dependent coagulation reactions [94,95], inhibit or impair thrombomodulin [96] and heparan sulfate [97] on vascular endothelial cells, suppress prostacyclin production from vascular endothelial cells that inhibits platelet aggregation [98], and increase the production and release of VWF [99,100] and plasminogen activator inhibitor [101]. Clinical findings are often found in deep-vein thrombosis, thrombocytopenia, recurrent miscarriage/stillbirth (infertility), cerebral infarction, migraine, and livedo [102]. APS is characterized by thrombosis in both arteries and veins.

Diagnosis of APS is based on laboratory findings and clinical findings. Laboratory findings include lupus anticoagulant (LA), anti-cardiolipin (CL) antibody IgG or IgM class, or anti-β2GPI antibody IgG or IgM class, detected at least twice with an interval of at least 12 weeks. The test results indicate that the patient is positive for antiphospholipid antibodies [103]. In addition, the clinical condition is positive if the patient has one or more arteriovenous thromboses that can be demonstrated on imaging or by pathological tests, or symptoms suggestive of thrombosis, such as complications of pregnancy including fetal death, premature birth, or three or more spontaneous miscarriages. The combination of positive laboratory and clinical findings allows for definitive diagnosis of APS [103].

Some patients with APS develop a consumptive decrease in platelet count due to platelet activation. Furthermore, as a characteristic of coagulation and fibrinolysis testing, APTT is prolonged in LA-positive cases [93]. FDP, D-dimer, and the coagulation activation marker TAT are elevated to varying degrees in the presence of active thrombosis [104]. In other words, if the platelet count is decreased and TAT, FDP, or D-dimer are increased, the possibility of thrombosis being present is high, and careful imaging tests are required. If LA-positive patients exhibit bleeding symptoms or prolonged PT, a decrease in plasma prothrombin activity should be considered. When LA positivity is combined with decreased plasma prothrombin activity, the diagnosis is lupus anticoagulant-hypoprothrombinemia syndrome [105].

Immunosuppressive therapy is used to treat catastrophic APS [106]. During the acute phase of APS, thrombolytic therapy and anticoagulant therapy are used in accordance with conventional thrombosis treatment [107]. Warfarin is used to prevent thrombosis in the chronic phase [106]. Although DOACs like warfarin are oral anticoagulants, their use for APS patients is inappropriate because these agents increase the risk of arterial thrombotic events [108]. In addition, patients who are aPL-positive but have no clinical symptoms usually receive careful follow-up [109], but low-dose aspirin or subcutaneous heparin injections are considered for pregnant women and post-surgical patients [109]. In triple-positive aPL (positive for LA, aCL, and aβ2GPI), which carry a high risk of thrombosis, the use of low-dose aspirin or heparin may be considered [109].

### 2.5. TMA

TMA is a syndrome characterized by the triad of thrombocytopenia, microangiopathic hemolytic anemia, and organ damage due to platelet thrombosis, resulting in platelet-based thrombi in microvessels throughout the body [110]. According to the etiology, TMA is classified into TTP, STEC-HUS, aHUS, secondary TMA, and other TMAs [10].

#### 2.5.1. TTP

TTP is a disease with five characteristics: decreased platelet count; hemolytic anemia; impaired renal function; fever; and neuropsychiatric symptoms [111]. However, less than 10% of cases have all of these symptoms [112]. A decrease in the activity of a disintegrin-like and metalloproteinase with thrombospondin type 1 motifs 13 (ADAMTS13), a VWF-cleaving enzyme, allows unusually large VWF multimers to form unusually large platelet thrombi in blood vessels, leading to ischemia-induced organ damage. Two types of TTP have been classified. The first is congenital TTP (Upshaw–Schulman syndrome), which is caused by a congenital abnormality in the gene for ADAMTS13. The second is acquired TTP (immune-mediated TTP), which is caused by autoantibodies against ADAMTS13 [113].

Diagnosis is usually performed by measuring ADAMTS13 activity when an unexplained decrease in platelet count or hemolytic anemia is observed, and cases in which the activity has significantly decreased to less than 10% of normal value are diagnosed as TTP [114]. Among these, if positive results are obtained for anti-ADAMTS13 autoantibody, acquired TTP (immune-mediated TTP) is diagnosed, and if the results are negative, congenital TTP is diagnosed [10]. However, in daily clinical practice, a definitive diagnosis of congenital TTP based on a negative autoantibody test alone is difficult to perform. ADAMTS13 activity should be measured over time to determine whether ADAMTS13 activity is consistently low. Congenital TTP is suspected when the ADAMTS13 activity of the parents is reduced to about 30–50% of normal value (suggesting heterozygous abnormality) [115,116], and the diagnosis is confirmed by genetic diagnosis [10].

The characteristics of coagulation and fibrinolysis tests in TTP have been reported [117,118]. Median coagulation and fibrinolysis test values reported in these reports were as follows: platelet count was significantly decreased to 9000/μL and 12,000/μL, PT-INR was within the normal range at 1.06 and 1.09 (reference value, 0.90–1.15), and APTT was within the normal range at 30 s and 31 s (reference value, 24.0–37.7 s). The fibrinogen level was 300 mg/dL and 310 mg/dL (reference value, 200–400 mg/dL), again within the normal range. On the other hand, FDP was 8.5 μg/mL and 16.7 μg/mL (reference value, <5.0 μg/mL), and D-dimer was 4.1 μg/mL and 7.1 μg/mL (reference value, <1.0 μg/mL), representing moderately high values. TAT level, as a marker of coagulation activation, was 9.6 ng/mL and 10.8 ng/mL (reference value, <3.9 ng/mL), and PIC, a fibrinolytic activation marker, was 1.7 and 2.0 μg/mL (reference value, <0.8 μg/mL), only slightly above the upper limit [117,118]. Because TTP and DIC often show similar findings from coagulation and fibrinolysis tests, differentiating between TTP and DIC based solely on coagulation and fibrinolysis test findings is challenging. Differentiating strategies that can be implemented in daily clinical practice are discussed later.

As for treatments for immune-mediated TTP, prompt and adequate plasma exchange [119,120,121] and corticosteroid therapy have been used [122]. Plasma exchange can remove unusually large VWF multimers, remove anti-ADAMTS13 antibodies, and replenish fresh ADAMTS13 [123]. However, concerns have been raised that supplementation with fresh ADAMTS13 may induce the further production of inhibitor and result in inhibitor boosting [124] and that approximately 20% of patients die due to thrombotic events such as myocardial infarction or cerebral infarction during the acute phase [119,125,126,127,128]. Treatment with steroids or plasma exchange alone is thus insufficient. Anti-CD20 antibody (rituximab), which suppresses the B lymphocytes that produce autoantibodies, has recently seen increasing use [129,130,131]. In addition, caplacizumab, an anti-VWF antibody, is effective in suppressing the onset of thrombosis in the acute phase. Although the use of caplacizumab improves platelet count recovery and shortens the time until plasma exchange discontinuation [132,133,134], ADAMTS13 antibodies may not be sufficiently removed due to a decrease in the number of plasma exchanges, resulting in delayed recovery of ADAMTS13 activity [135]. The utility of antiplatelet drugs for TTP is unclear [136]. Platelet transfusions are generally not performed unless bleeding is considered potentially fatal, given the risk of exacerbating thrombosis [136,137,138]. Other reports include cyclophosphamide [139], vincristine [140], cyclosporine [141], splenectomy [142], and high-dose immunoglobulin therapy [143], but the usefulness of these treatments remains unclear and are not the standard of care.

In congenital TTP, supplementation with fresh frozen plasma (FFP) is the most common treatment [144]. Some cases require regular infusions of FFP, and others require FFP infusions only when TTP worsens. The frequency of effective FFP infusions and the optimal activity level of ADMATS13 activity are unclear [145]. In addition, infection, pregnancy, and alcohol increase the expression of VWF [146] but not ADAMTS13 production. ADAMTS13 therefore exhibits a relative decrease with respect to VWF, which is a factor that induces TTP, so caution is required. In recent years, the efficacy of recombinant ADAMTS13 preparations against congenital TTP has received attention [147,148,149]. Higher activity levels of ADAMTS13 can be maintained using recombinant ADAMTS13 rather than regular prophylactic FFP supplementation. This will hopefully not only reduce the number of platelets and the incidence of TTP, but also improve long-term organ damage caused by microthrombi.

#### 2.5.2. STEC-HUS

STEC-HUS is HUS caused by enterohemorrhagic *E. coli* (EHEC) infection. EHEC is also called Shiga toxin-producing *E. coli* (STEC), and the associated HUS is thus commonly referred to as STEC-HUS. Shiga toxin binds to the globotriosyceramide 3 receptor (Gb3 receptor) of target organs such as the brain, kidneys, heart, and pancreas. Shiga toxin is taken into cells and is cytotoxic by cleaving specific parts of ribosomes and inhibiting protein synthesis [150]. Gb3 receptors are thought to be abundantly expressed in the vascular endothelial cells of the renal glomerulus and renal tubules [150], greatly increasing the susceptibility to renal damage. STEC is thought to not only damage vascular endothelial cells but also reduce vascular endothelial cell function, leading to the overproduction of inflammatory cytokines and chemokines and inducing excessive coagulation activation [150].

STEC-HUS is diagnosed from the following three main symptoms: hemolytic anemia (anemia accompanied by increased lactate dehydrogenase (LDH) and indirect bilirubin and decreased haptoglobin), decreased platelet count, and acute kidney injury. In addition, a definitive diagnosis of EHEC infection is performed from the isolation culture of EHEC and tests for Shiga toxin, fecal O157 antigen, and serum O157LPS antibody.

Few reports have discussed the characteristics of coagulation and fibrinolysis markers in STEC-HUS [151,152,153]. PT and APTT are normal to mildly prolonged, fibrinogen is within the normal range, FDP and D-dimer are elevated, and TAT, a marker of coagulation activation, presents as elevated. The accumulation of more cases is needed to better characterize this pathology.

Treatment strategies for STEC-HUS are fluid therapy, blood transfusion, antihypertensive therapy, dialysis, plasmapheresis, and anticoagulants. The infusion of fluids is recommended to prevent the onset of acute kidney injury and dialysis. Because overload may occur during the oliguric and anuric phases after the onset of HUS, the daily infusion volume should be determined based on urine volume, fluid loss by insensible excretion, and fecal volume [154,155]. In addition, red blood cell transfusion is recommended for anemia with hemoglobin (Hb) ≤ 6.0 g/dL [156]. Platelet transfusions are generally contraindicated because of the risk of promoting the formation of microthrombi [157]. Hypertension is a frequent complication and causes acute heart failure and posterior reversible encephalopathy syndrome [158], so prompt antihypertensive therapy is necessary. Calcium blockers are the first choice, but if the effect is insufficient, renin–angiotensin system inhibitors are also used [158]. Dialysis therapy should be considered when symptoms such as oliguria, uremic symptoms, or hyperkalemia are observed and do not respond to medication. Anticoagulant therapy is not recommended for HUS without DIC. If DIC is present, agents such as nafamostat or recombinant thrombomodulin may be used [158]. Furthermore, conservative treatment is the main treatment for EHEC infections, and no consensus has been reached regarding the use of antibiotics [159]. Administration of antidiarrheal agents is not recommended [160,161,162].

#### 2.5.3. aHUS

Originally, aHUS was a broad concept that referred to TMA excluding TTP and STEC-HUS [163]. However, it has gradually become clear that abnormalities in complement-related genes contribute to the onset of aHUS, so aHUS has come to be distinguished from secondary TMAs and other TMAs, which are discussed later [164]. Currently, aHUS refers to TMA excluding TTP, STEC-HUS, and cases with an obvious cause as secondary TMA. aHUS can be interpreted as TMA caused by complement abnormalities. Mutations in complement-related genes result in abnormal activation of the alternative complement pathway [165]. C5a produced from an abnormally activated alternative complement pathway acts on leukocytes to express tissue factor and induce a coagulation reaction [166], and C5b-9 acts on activated platelets and activated endothelial cells. Phosphatidylserine is exposed on the outside of the cell membrane of endothelial cells, on which the prothrombinase complex is formed, promoting the coagulation reaction and producing large amounts of thrombin [167]. Due to these mechanisms, aHUS is a disease that causes thrombosis and associated organ damage, particularly in the form of acute kidney injury.

Diagnosis of aHUS is divided into clinical diagnosis and definitive diagnosis. For clinical diagnosis, when the three symptoms of microangiopathic hemolytic anemia, decreased platelet count, and acute kidney injury are observed, and TTP, STEC-HUS, and secondary TMA can be ruled out, aHUS is suspected and intervention should be considered. For a definitive diagnosis, some reports recommend measuring blood C3, C4, factor H, factor I, and factor B as well as analyzing the expression level of CD46 on leukocytes [168]; however, this alone does not necessarily lead to a definitive diagnosis. Accurate and definitive diagnosis requires searching for known gene mutations (CFH, CFB, CFI, C3, CD46, THBD, DGKE, PLF) and determining the presence or absence of anti-factor H antibodies. However, it takes several weeks to obtain test results, and treatment should be based on the clinical diagnosis. Delayed initiation of treatment may result in irreversible renal failure [110,164]. In addition, aHUS may be caused by genes other than those listed above, and even if a gene search is performed, a definitive diagnosis may not always be reached [169].

Characteristics of coagulation and fibrinolysis tests are thought to be decreased platelet count, elevated FDP, D-dimer levels reflecting the presence of thrombus formation, and elevated TAT reflecting excessive coagulation activation, but no reports have provided a detailed analysis.

Plasma exchange and plasma infusion have been used until recently to treat aHUS [170]. These treatments aim to reduce complement activation by replenishing normal complement-related proteins and removing abnormal complement-related proteins and anti-factor H antibodies. In recent years, the anti-C5 antibody agents eculizumab and ravulizumab have been used [171,172,173,174,175,176]. Although the use of these agents has the potential to improve not only platelet counts but also overall survival and renal prognosis, the risk of fatal meningococcal infection [177,178] needs to be determined on a case-by-case basis.

#### 2.5.4. Secondary TMA and Other TMAs

Among TMAs, those caused by some of the underlying diseases mentioned below are categorized as secondary TMA; TMA with no known cause is classified as other TMAs.

Causes of secondary TMA include autoimmune diseases or collagen diseases (e.g., systemic lupus erythematosus, systemic sclerosis, antiphospholipid syndrome) and infectious diseases (e.g., streptococcal toxic shock syndrome, human immunodeficiency virus, influenza virus, hepatitis C virus, cytomegalovirus, pertussis, varicella, rickettsia [179], invasive pneumococcal disease) [180]. However, differentiating between aHUS and secondary TMA is difficult as aHUS may manifest in patients who have a predisposition to aHUS due to infection [181]. Furthermore, it should be noted that TMA caused by Pneumococci is exacerbated by FFP infusion. When FFP is administered for TMA caused by pneumococci, Thomsen–Friedenreich antigens are exposed on the surface of red blood cells, platelets, and renal glomerular endothelial cells by neuraminidase produced by pneumococci. Anti-Thomsen–Friedenreich IgM antibodies abundant in plasma react with the antigen, and TMA worsens. Plasma therapy for TMA due to pneumococcal infection is therefore contraindicated [182]. Causes of drug-induced TMA include antiplatelet drugs (ticlopidine, clopidogrel), antiparasitic drugs (quinine), antiviral drugs, interferons, anticancer drugs (gemcitabine), immunosuppressive drugs (cyclosporine, tacrolimus, sirolimus), viral vectors (abeparvovec), proteasome inhibitors (carfilzomib), narcotics, and oral contraceptives [183]. Antiplatelet drugs such as ticlopidine and clopidogrel, interferon, immune checkpoint inhibitors, and COVID-19 vaccines can significantly reduce ADAMTS13 activity after administration, resulting in an inhibitor of ADAMTS13 leading to TTP [184]. The course of drug-induced TTP differs from that of immune-mediated TTP in that it often resolves spontaneously simply with discontinuation of the suspected drug [183]. Other causes of secondary TMA include pregnancy, hypertensive emergencies, malignancies, hematopoietic stem cell or renal transplantation, and abnormal cobalamin C metabolism [185]. In particular, in the end-of-life stage of malignant tumors, not only TMA but also DIC are often present, making differentiation between the two difficult.

Other TMAs are diagnosed when a patient presents with TMA but not with an obvious underlying disease. Specifically, other TMAs are categorized when STEC-HUS and TTP are not suspected, no underlying disease that could cause TMA is present, and complement-related factor abnormalities also show negative results. The presence of unknown genetic pathological variants and background pathologies that produce TMAs may be latent, and further research is warranted.

### 2.6. DIC

DIC is a condition in which multiple microthrombi occur in small blood vessels due to systemic persistent and marked activation of coagulation in the presence of an underlying disease [59]. Sepsis, solid tumors, and hematopoietic malignancies such as acute leukemia are the three most common underlying diseases. DIC is also known to occur with tissue damage from trauma, burns, heat stroke, aortic aneurysms and vascular malformations [186], and obstetrical complications (placental abruption, amniotic fluid embolism) [187]. Although marked activation of coagulation is a common pathology in DIC, the degree of fibrinolysis activation varies greatly depending on the underlying disease [57,58,59,188]. Sepsis in which fibrinolytic activation is suppressed by plasminogen activator inhibitor 1 (PAI-1) results in a suppressed-fibrinolytic-type DIC, with ischemic organ damage due to microthrombosis. Acute promyelocytic leukemia, aortic aneurysms, and vascular malformations, in which fibrinolytic activation is excessively enhanced, are associated with the enhanced-fibrinolytic-type DIC, with bleeding symptoms. Balanced-fibrinolytic-type DIC is commonly seen in patients with solid tumors, which is characterized by an equilibrium between coagulation activation and fibrinolysis activation and often does not present with clear symptoms except in advanced cases [57,58,59,188]. The differences in laboratory test results between different types of DIC are summarized in Table 1.

The presence of underlying disease is essential for diagnosing DIC, and a scoring system consisting of multiple coagulation tests is used to reach the diagnosis [186,189,190,191]. The scoring systems for DIC diagnosis are shown in Table 2. In some cases, DIC is first diagnosed based on abnormal laboratory values such as decreased platelet counts or elevated D-dimer levels, and aortic aneurysms or malignant tumors are discovered as secondary phenomena through a detailed examination of the disease underlying DIC. On the other hand, there are conditions that present abnormalities of coagulation test results similar to DIC [186], so attention must be paid to the interpretation of laboratory test results.

The characteristics of coagulation/fibrinolysis tests in DIC subtypes are shown in Table 1. Notably, in enhanced-fibrinolytic-type DIC, many cases show PT and APTT within the normal range [57,58]. PT and APTT alone therefore cannot diagnose or exclude DIC. TAT, prothrombin fragment 1 + 2 (F1 + 2), and soluble fibrin (SF), which reflect coagulation activation as the essential component of DIC, should be always tested [186]. PIC, plasminogen, and α_2_PI are also important tests not only for evaluating the degree of fibrinolysis activation but also for adjusting the doses of antifibrinolytic therapies given in combination with anticoagulation [57,58,186]. Specifically, the dose of anticoagulants is adjusted by the coagulation activation marker TAT, while the dose of antifibrinolytic drugs is adjusted by the fibrinolysis activation marker PIC [57,58]. Another characteristic of coagulation studies for DIC is a marked decrease in factor XIII activity [192]. Factor XIII is involved in wound healing and hemostatic clot stabilization by incorporating fibronectin, which contributes to tissue repair, and α_2_PI, an inhibitor of fibrinolysis, into fibrin clots [193]. The decreased activity of factor XIII in DIC may result from consumption due to excessive activation of coagulation [194]. Factor XIII preparations have been highlighted as a hemostatic treatment option for enhanced-fibrinolytic-type DIC presenting with hemorrhage [195,196].

The cornerstone of DIC treatment is to treat the underlying disease. However, treatment of the underlying disease is difficult in many cases. For example, aortic aneurysms [57], vascular malformations [197], and end-of-life-stage malignancy [198] are sometimes difficult to treat. The presence of DIC may also prevent safe treatment. For example, aortic aneurysm surgery in the presence of enhanced-fibrinolytic-type DIC carries a risk of fatal bleeding.

In addition, if DIC is expected to improve with treatment of the underlying disease, or if the patient has no symptoms of DIC and is unlikely to experience exacerbation of DIC, follow-up without DIC treatment is an appropriate option [57,58]. Unfractionated heparin, low molecular weight heparin, nafamostat, and thrombomodulin [57,58] are commonly used as anticoagulation therapy in DIC. For long-term outpatient control of DIC, DOACs can also have a dramatic effect on DIC [69,197,199,200,201,202,203,204,205,206,207,208,209]. Warfarin is also an anticoagulant, but its use in DIC is contraindicated because this agent aggravates DIC [69]. All anticoagulants effective against DIC inhibit active coagulation factors, but warfarin decreases coagulation factors as substrates, so active coagulation factors already present in the body cannot be suppressed. By suppressing coagulation factors as substrates, warfarin not only fails to suppress coagulation activation already present in the body but also suppresses the production of the coagulation factors essential for the organism.

Replacement therapy includes the replacement of coagulation factor with fresh-frozen plasma and concentrated platelet, as well as factor XIII preparations for DIC bleeding [57,58].

In enhanced-fibrinolytic-type DIC, anticoagulation and antifibrinolytic therapy may be used in combination [57,58]. The use of antifibrinolytic agents alone is contraindicated because of the risk of inducing fatal thrombosis [210,211]. Dose adjustment is difficult when anticoagulation and antifibrinolytic therapy [57,58] are combined, and consultation with a specialist is recommended.

## 3. How to Distinguish Thrombosis with Thrombocytopenia Syndrome

Many diseases categorized as TTS are important, and differential diagnosis can be difficult; furthermore, several scoring systems have been in use for a long time. In this section, we discuss useful methods for differentiating the aforementioned TTS based on scoring systems.

### 3.1. French Score

The French score was developed to identify TTP with reduced ADAMTS13 activity among TMA patients with hemolytic anemia and platelet counts <150,000/μL, schistocytes in peripheral blood smears, and a negative Coombs test [212].

- Platelet count <30,000/μL;
- Serum creatinine <200 μmol/L (<2.26 mg/dL);
- Positive results for antinuclear antibody.

When one or more of these three criteria are met, the sensitivity to detect decreased ADAMTS13 activity is 98.8%, the specificity is 48.1%, the positive predictive value is 85.0%, and the negative predictive value is 93.3%. When all three criteria are met, the sensitivity is 46.9%, the specificity is 98.1%, the positive predictive value is 98.7%, and the negative predictive value is 38.6%.

TTP resulting from decreased ADAMTS13 activity is associated with more severe thrombocytopenia than non-TTP TMA, but the renal involvement is often mild [126,212]. French scores comprise items that can be measured quickly in daily clinical practice and are very easy to use, but the difficulty is that high sensitivity and specificity cannot be achieved at the same time.

### 3.2. PLASMIC Score

The PLASMIC score was developed to identify TTP with ADAMTS13 activity <10% among TMA patients with platelet counts <150,000/μL and schistocytes in peripheral blood smears [213].

- Platelet count <30,000/μL;
- Serum creatinine <2.0 mg/dL;
- Hemolytic findings (indirect bilirubin >2 mg/dL or reticulocytes >2.5% or haptoglobin detection sensitivity <2.5%);
- No active cancer;
- No history of solid-organ or stem cell transplantation;
- Mean corpuscular volume (MCV) <90 fL;
- PT-INR < 1.5.

When 6 or 7 of the above 7 criteria are met, the likelihood of ADAMTS13 activity <10% is 62–82% as compared with a likelihood of 5–24% for 5 criteria and 0–4% for 0–4 criteria, making this scoring system a very accurate method for identifying groups with reduced ADAMTS13 activity among TMAs. However, serum creatinine tends to be higher in elderly TTP patients, which tends to underestimate the PLASMIC score and lead to false negatives [214]. Megaloblastic anemia tends to present with macrocytic anemia and high MCV but can be misdiagnosed as TTP because of low platelet counts and hemolytic findings due to ineffective hematopoiesis [215]. Careful interpretation is therefore required.

### 3.3. Scoring System for Differentiating TTP and Septic DIC

Clinically distinguishing TTP from septic DIC is difficult. Thrombocytopenia, renal dysfunction, fever, and unconsciousness are present with TTP and DIC. Distinguishing between the two using existing scoring systems is also very difficult. For example, approximately 30% of TTP patients meet the DIC diagnostic criteria set by the International Society on Thrombosis and Haemostasis, and 70% of DIC patients meet one or more items of the French score [118]. Clearly differentiating between the two using the PLASMIC score is difficult.

Measurement of ADAMTS13 activity is important for differentiating between TTP and DIC, but the number of facilities that can perform such testing rapidly is limited. One report examined the distinction between TTP and DIC using coagulation tests [117]. This report showed that the TTP group had significantly lower platelet counts, prolonged PT and APTT, decreased AT activity, and decreased FDP and D-dimer compared with the DIC group. This can be interpreted as resulting from the fact that TTP primarily involves platelet thrombus and coagulation activation as a secondary event, whereas in DIC, fibrin thrombus due to coagulation activation is the primary pathophysiology [216].

Diagnosing or excluding TTP is difficult when the PLASMIC score is four or five [217]. Such cases account for about 60% of DIC cases and 25% of TTP cases [217]. In such cases, a clinical diagnosis of TTP can be performed if the LDH/Hb ratio is ≥53.7 with a sensitivity of 0.94 and specificity of 0.91. Strong hemolysis results in increased LDH levels and decreased Hb levels. The LDH/Hb ratio is thought to represent an indicator reflecting higher levels of hemolysis in TTP compared with DIC.

**Figure 2:**
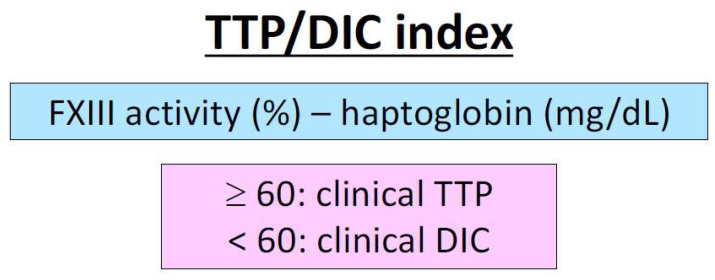
TTP/DIC index. In patients where there is difficulty differentiating between TTP or DIC, factor XIII activity and haptoglobin can be measured to obtain an index by subtracting haptoglobin (mg/dL) from FXIII activity (%). If the index is ≥60, TTP is clinically suspected. If the index is <60, DIC is clinically suspected. FXIII activity alone or haptoglobin alone are also useful for distinguishing between TTP and DIC. The cutoff level of haptoglobin is 2.868 mg/dL, above which DIC is suspected and below which TTP is suspected (AUC 0.832 (95% confidence interval, 0.727–0.938)). The cut-off level of factor XIII activity is 76.0%, above which TTP is suspected and below which DIC is suspected (AUC 0.931 (95% confidence interval, 0.873–0.989)) [118]. Abbreviations: TTP, thrombotic thrombocytopenic purpura; DIC, disseminated intravascular coagulation; AUC, area under the curve.

We examined the differentiation between TTP and septic DIC based on the localization of the thrombus formation and the degree of coagulation activation [118]. In this report, we subtracted haptoglobin (mg/dL) from factor XIII activity (%) and considered TTP present for values ≥ 60 and DIC for values < 60 (Figure 2). In DIC, where excessive coagulation activation occurs, factor XIII activity is decreased. In TTP, where thrombosis occurs in fast-flowing arterioles, haptoglobin is highly decreased, reflecting the intensity of hemolysis. On the other hand, in DIC, the production of haptoglobin, an inflammatory protein, is increased [218,219], and the site of thrombus formation is in small veins with slow blood flow. The sensitivity and specificity of the index are high at 94.3% and 86.7%, respectively, and both factor XIII activity and haptoglobin alone offer high sensitivity and specificity. We call this evaluation index the TTP/DIC index [118]. Decreased factor XIII activity has been observed in other types of DIC [192], and haptoglobin is thought to be decreased in any type of TMA as in TTP because of the strong hemolysis due to platelet thrombus. The scope of the study of FXIII activity and haptoglobin is expected to be further expanded upon and is expected to upgrade the TMA/DIC index.

### 3.4. Differentiation of TTS in Daily Clinical Practice Utilizing Scoring Systems

**Figure 3:**
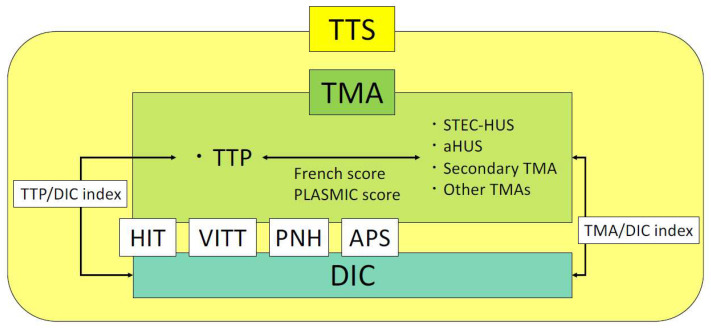
Relationship and differentiation of diseases among TTS. Of the diseases included in TTS, platelet thrombosis due to platelet activation is the main cause of TMA and fibrin thrombosis due to coagulation activation is the main cause of DIC. HIT, VITT, PNH, and APS may have both TMA and DIC features. The TTP/DIC index is used to differentiate TTP from DIC. Further studies will hopefully expand on the TMA/DIC index to distinguish between TMA and DIC. Abbreviations: TTS, thrombosis with thrombocytopenia syndrome; TMA, thrombotic microangiopathy; DIC, disseminated intravascular coagulation; HIT, heparin-induced thrombocytopenia; VITT, vaccine-induced immune thrombotic thrombocytopenia; PNH, paroxysmal nocturnal hemoglobinuria; APS, antiphospholipid antibody syndrome; TTP, thrombotic thrombocytopenic purpura; STEC-HUS, Shiga toxin-producing *Escherichia coli*-associated hemolytic uremic syndrome; aHUS, atypical hemolytic uremic syndrome.

We have described the pathogenesis of each disease categorized as TTS, test characteristics, and indices of differentiation already in use. The relationships between each disease are shown in Figure 3. The keywords leading to a suspicion of each disease categorized as TTS and important tests for reaching a definitive diagnosis are summarized in Table 3.

## 4. Summary

For many of the diseases categorized as TTS, it is difficult to understand the individual pathophysiology, assemble and interpret the necessary tests for differentiation, and perform a definitive diagnosis. Delayed diagnosis can affect patient prognosis. Scoring systems have the ability to overcome both of these problems. Based on the physical examinations and general laboratory findings, we would like to enumerate the diseases that should be differentiated and strive for prompt definitive diagnosis and therapeutic interventions based on the implementation of appropriate tests and scoring systems.

## Figures and Tables

**Table 1:** DIC types and characteristics of laboratory tests.

Classification	Suppressed- Fibrinolytic-Type	Balanced- Fibrinolytic-Type	Enhanced- Fibrinolytic-Type
Underlying disease	Sepsis	Solid tumors	APL, aortic aneurysm, severe COVID-19
Platelet count	Decreased	Decreased	Decreased
PT	Prolonged	Prolonged	Normal to prolonged
APTT	Prolonged	Prolonged	Mildly shortened to prolonged
Fibrinogen	Normal to increased	Decreased	Markedly decreased
FDP	Mildly increased	Increased	Markedly increased
D-dimer	Mildly increased	Increased	Increased
FDP/D-dimer ratio	Approximately 1	Approximately 1–2	Approximately 2–5
Antithrombin	Decreased	Decreased to normal	Normal
TAT (coagulation activation marker)	Markedly increased	Markedly increased	Markedly increased
PIC (fibrinolysis activation marker)	Mildly increased	Increased	Markedly increased
α_2_PI	Normal	Mildly decreased	Markedly decreased
Plasminogen	Decreased	Mildly decreased	Decreased
PAI	Markedly increased	Mildly increased	Normal to mildly increased

Abbreviations: APL, acute promyelocytic leukemia; COVID-19, coronavirus disease 2019; PT, prothrombin time; APTT, activated partial thromboplastin time; FDP, fibrin/fibrinogen degradation products; TAT, thrombin–antithrombin complex; PIC, plasmin-α_2_ plasmin-inhibitor complex; α_2_PI, α_2_-plasmin inhibitor; PAI-1, plasminogen activator inhibitor 1.

**Table 2:** Comparison of DIC diagnostic criteria.

Criteria	JMHLW	ISTH	JAAM	JSTH		
				Basic	Hematopoietic Disorder	Infectious
Platelet count (×10^4^/μL)	≤12 + 1 pt ≤8 + 2 pt ≤5 + 3 pt	≤10 + 1 pt ≤5 + 2 pt	≤12 + 1 pt ≤8 + 3 pt >30% reduction/24 h + 1 pt >50% reduction/24 h + 3 pt	>8, ≤12 + 1 pt >5, ≤8 + 2 pt ≤5 + 3 pt ≥30% decrease/24 h + 1 pt	-	>8, ≤12 + 1 pt >5, ≤8 + 2 pt ≤5 + 3 pt ≥30% decrease/24 h + 1 pt
PT ratio	≥1.25 + 1 pt ≥1.67 + 2 pt	-	≥1.2 + 1 pt	≥1.25, <1.67 + 1 pt ≥1.67 + 2 pt		
PT (s)	-	Prolonged PT ≥3 s + 1 pt ≥6 s + 2 pt	-	-		
Fibrinogen (mg/dL)	≤150 + 1 pt ≤100 + 2 pt	≤100 + 1 pt	-	>100, ≤150 + 1 pt ≤100 + 2 pt		-
FDP (μg/mL)	≥10 + 1 pt ≥20 + 2 pt ≥40 + 3 pt	≥10 + 2 pt ≥25 + 3 pt	≥10 + 1 pt ≥25 + 3 pt	≥10, <20 + 1 pt ≥20, <40 + 2 pt ≥40 + 3 pt		
Antithrombin (%)	-	-	-	≤70% + 1 pt		
TAT, SF, F1 + 2	-	-	-	≥2-fold upper limit of normal + 1 pt		
SIRS	-	-	≥3 + 1 pt	-		
Underlying diseases	+1 pt	-	-	-		
Bleeding	+1 pt	-				
Organ failure	+1 pt					
Liver failure	-	-	-	Yes -3 pt		
Diagnosis of DIC	≥7 pt	≥5 pt	≥4 pt	≥6 pt	≥4 pt	≥5 pt

Abbreviations: DIC, disseminated intravascular coagulation; PT, prothrombin time; FDP, fibrin/fibrinogen degradation products; TAT, thrombin–antithrombin complex; SF, soluble fibrin; F1 + 2, prothrombin fragment 1 + 2; SIRS, systemic inflammatory response syndrome; JMHLW, Japanese Ministry of Health, Labour and Welfare; ISTH, International Society on Thrombosis and Haemostasis; JAAM, Japanese Association of Acute Medicine; JSTH, Japanese Society on Thrombosis and Hemostasis.

**Table 3:** Points of differentiation and definitive diagnosis of TTS.

Disease		Important Clinical and Laboratory Findings	
HIT		History of exposure to heparin, 4Ts score, anti-HIT antibody	
VITT		Vaccination history, thrombosis at unusual sites, anti-HIT antibodies (ELISA assay)	
PNH		Hemolysis, hemoglobinuria, PNH cells (CD55/59-negative)	
APS		Repeated arteriovenous thrombosis, prolonged APTT, positive for at least one of LA/anti-CL antibody/anti-β2GPI antibody	
TMA	TTP	Schizocyte, marked decrease in haptoglobin	Marked decrease in ADAMTS13 activity
	STEC-HUS		Bloody stool with mucous, EHEC infection, Shiga toxin
	aHUS		Abnormalities in complement-related genes
	Secondary TMA, other TMAs		Underlying disease, other TMAs ruled out
DIC		PT, APTT, fibrinogen, FDP, D-dimer, antithrombin, TAT, PIC, plasminogen, α_2_PI	

The diagnostic criteria and classification of DIC are referred to in Table 1 and Table 2. Abbreviations: TTS, thrombosis with thrombocytopenia syndrome; HIT, heparin-induced thrombocytopenia; VITT, vaccine-induced immune thrombotic thrombocytopenia; PNH, paroxysmal nocturnal hemoglobinuria; APS, antiphospholipid antibody syndrome; TMA, thrombotic microangiopathy; TTP, thrombotic. thrombocytopenic purpura; STEC-HUS, Shiga toxin-producing *Escherichia coli*-associated hemolytic uremic syndrome; aHUS, atypical hemolytic uremic syndrome; DIC, disseminated intravascular coagulation; ELISA, enzyme-linked immunosorbent assay; PT, prothrombin time; APTT, activated partial thromboplastin time; FDP, fibrin/fibrinogen degradation products; TAT, thrombin–antithrombin complex; PIC, plasmin-α_2_-plasmin-inhibitor complex; α_2_PI, α_2_-plasmin inhibitor; CD, cluster of differentiation; LA, lupus anticoagulant; GPI, glycoprotein I; ADAMTS13, a disintegrin-like and metalloproteinase with thrombospondin type 1 motifs 13; EHEC, enterohemorrhagic *Escherichia coli.*
